# Right Ventricular Dysfunction in Patients Experiencing Cardiotoxicity during Breast Cancer Therapy

**DOI:** 10.1155/2015/609194

**Published:** 2015-08-03

**Authors:** Anna Calleja, Frédéric Poulin, Ciril Khorolsky, Masoud Shariat, Philippe L. Bedard, Eitan Amir, Harry Rakowski, Michael McDonald, Diego Delgado, Paaladinesh Thavendiranathan

**Affiliations:** ^1^Division of Cardiology, Peter Munk Cardiac Center, Toronto General Hospital, University Health Network, University of Toronto, 200 Elizabeth Street, Toronto, ON, Canada M5G 2C4; ^2^Division of Cardiology, Hôpital du Sacré-Coeur de Montréal, University of Montreal, 5400 Boulevard Gouin Ouest, Montreal, QC, Canada H4J 1C5; ^3^Division of Medical Imaging, Peter Munk Cardiac Center, Toronto General Hospital, University Health Network, University of Toronto, 200 Elizabeth Street, Toronto, ON, Canada M5G 2C4; ^4^Division of Medical Oncology & Hematology, Princess Margaret Cancer Center, University Health Network, University of Toronto, 610 University Avenue, Toronto, ON, Canada M5T 2M9

## Abstract

*Background*. Right ventricular (RV) dysfunction during cancer therapy related cardiotoxicity and its prognostic implications have not been examined. *Aim*. We sought to determine the incidence and prognostic value of RV dysfunction at time of LV defined cardiotoxicity. *Methods*. We retrospectively identified 30 HER2+ female patients with breast cancer treated with trastuzumab (± anthracycline) who developed cardiotoxicity and had a diagnostic quality transthoracic echocardiography. LV ejection fraction (LVEF), RV fractional area change (RV FAC), and peak systolic longitudinal strain (for both LV and RV) were measured on echocardiograms at the time of cardiotoxicity and during follow-up. Thirty age balanced precancer therapy and HER2+ breast cancer patients were used as controls. *Results*. In the 30 patients with cardiotoxicity (mean ± SD age 54 ± 12 years) RV FAC was significantly lower (42 ± 7 versus 47 ± 6%, *P* = 0.01) compared to controls. RV dysfunction defined by global longitudinal strain (GLS < −20.3%) was seen in 40% (*n* = 12). During follow-up in 16 out of 30 patients (23 ± 15 months), there was persistent LV dysfunction (EF < 55%) in 69% (*n* = 11). Concomitant RV dysfunction at the time of LV cardiotoxicity was associated with reduced recovery of LVEF during follow-up although this was not statistically significant. *Conclusion*. RV dysfunction at the time of LV cardiotoxicity is frequent in patients with breast cancer receiving trastuzumab therapy. Despite appropriate management, LV dysfunction persisted in the majority at follow-up. The prognostic value of RV dysfunction at the time of cardiotoxicity warrants further investigation.

## 1. Introduction

Breast cancer is the leading cause of cancer in women worldwide [1, 2]. Survival from breast cancer has improved significantly over the past 15–20 years primarily due to advances in cancer treatment [3]. However, many anticancer drugs used for the treatment of patients with breast cancer have the potential to cause cardiac toxicity (cardiotoxicity). Anthracycline-based chemotherapy and trastuzumab (TZM), a monoclonal antibody against the HER2 receptor, are of particular concern due to the high incidence of cardiotoxicity individually and with combined use [4, 5]. Once left ventricular (LV) dysfunction or heart failure (HF) occurs from anthracycline and/or TZM based therapy the prognosis can be poor with lack of LV function recovery in up to 40–58% of the patients and subsequent major adverse cardiac events [6, 7]. Due to the poor prognosis of advanced cardiac dysfunction [6, 8], many argue for efforts to identify early cardiac dysfunction so that appropriate intervention can be initiated to prevent HF [9]. This can include administration of cardiac treatment such as beta-blockers, ACE inhibitors, and dexrazoxane; selection of alternative cancer regimens or dose adjustment; and transient cessation of cancer treatment [9]. In TZM treated patients in particular, early cardiac dysfunction is identified by repeated cardiac imaging performed prior and during cancer therapy. Cardiotoxicity is commonly defined based on a symptomatic fall in LVEF of >5 percentage points or an asymptomatic fall of >10 percentage points to <55% between pre- and during treatment measurements as defined by the cardiac review and evaluation committee criteria (CREC) [10].

To date, there has been very little focus on the toxic effects of cancer therapy on the right ventricle (RV) [11, 12]. Given the thinner structure of the RV with fewer myofibrils, the RV may also be susceptible to damage by cardiotoxic therapy. Several studies have shown that RV wall motion abnormalities [13] or functional abnormalities [12, 14] occur* during cancer therapy*; however, this finding has not been universally observed [11, 15]. The presence of RV dysfunction at the* time of LV cardiotoxicity* and whether it has prognostic implications has not been examined. However, in many other cardiovascular diseases the concomitant RV dysfunction is associated with worse outcomes [16–18]. In this study we sought to determine the incidence of RV dysfunction at the time of cardiotoxicity in women with HER2+ breast cancer receiving treatment with trastuzumab using measurements of fractional area change and myocardial peak systolic longitudinal strain. Secondly as a hypothesis generating objective we examined the prognostic value of RV dysfunction in subsequent LV function recovery during follow-up in a subgroup of patients who had follow-up imaging after completion of cancer therapy.

## 2. Methods

### 2.1. Study Population

We retrospectively identified all women >18 years of age with HER2/neu overexpressing (HER2+) breast cancer of any stage treated with TZM with or without anthracyclines at a large cancer referral center (Princess Margaret Cancer Center, Toronto, Canada) between 2006 and 2013 from the hospital pharmacy database. We included patients who (1) developed cardiotoxicity during the treatment course using the CREC criteria [10] and (2) had an echocardiogram with adequate image quality at the time of diagnosis of cardiotoxicity. For each patient the following data were obtained through electronic patient records: patient demographics, cardiac risk factors, previous cardiac history, cardiac medication use, cancer history, cancer therapy with doses used, radiotherapy history, LVEF and RVEF measurements by multigated acquisition (MUGA) pre-cancer therapy and at the time of cardiotoxicity, clinical symptoms of heart failure, and management of cardiotoxicity. The study protocol was approved by the institutional Research Ethics Board.

### 2.2. Controls

Age and cardiac risk factor balanced women with a diagnosis of HER2+ breast cancer without history of any previous cardiovascular disease and who had an echocardiogram prior to initiation of any cancer therapy were included as controls. This was necessary as currently existing normal values for some of the myocardial function measures used in this study such as RV strain are variable and age- and vendor-specific and have never been studied in patients with cancer. Also during the study period it was routine for patients at our center to be followed by MUGA scans as opposed to echocardiography. Therefore, since baseline echocardiography was not available in our patients with cardiotoxicity this comparison with a control group was essential.

### 2.3. Transthoracic Echocardiogram: Conventional Parameters and Myocardial Strain

Echocardiography studies from patients at the time of LV cardiotoxicity and from the control group were read together blinded to the group designation and clinical history. LVEF was calculated using the Bi-plane Simpson's method and RV FAC (analogous to LVEF) following existing American Society of Echocardiography (ASE) guidelines [19, 20].

**Figure 1:**
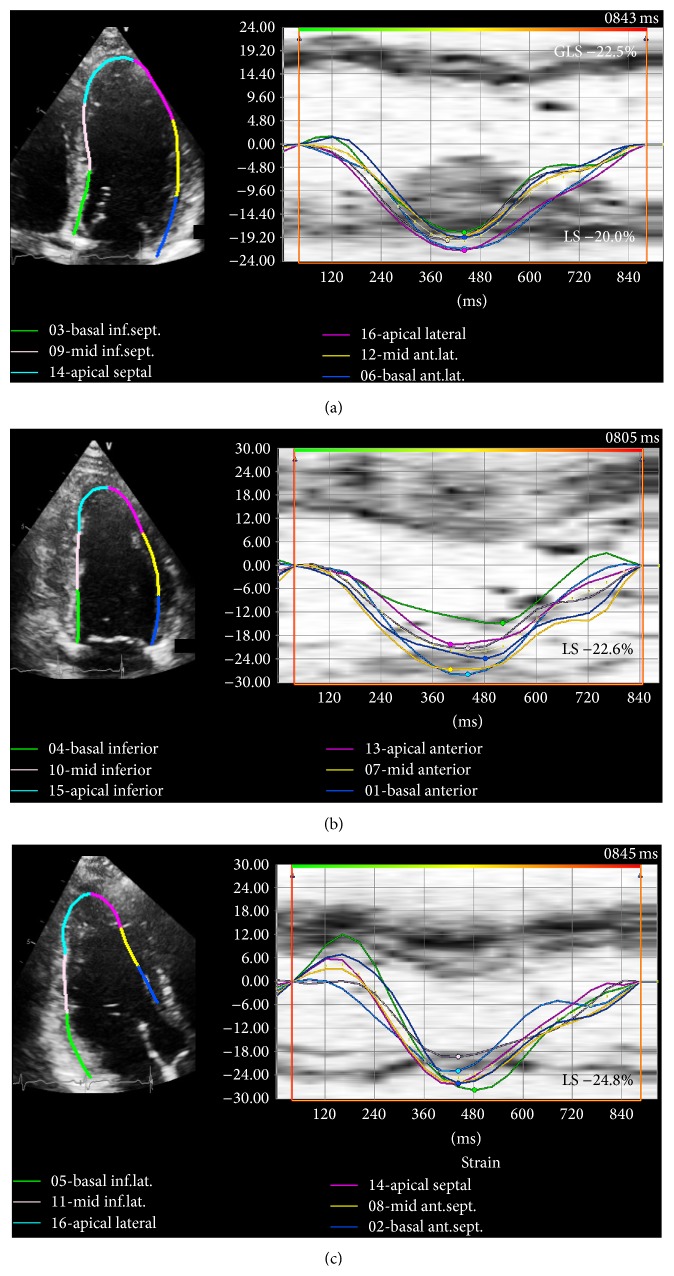
Left ventricular peak systolic longitudinal strain. Representative example of normal left ventricular peak endocardial strain curves: left panel shows B-mode images with endocardial tracings in the (a) 4-chamber, (b) 2-chamber, and (c) 3-chamber views with their corresponding longitudinal strain curves to its right. For each view 5-6 curves are shown representing strain values for each of the myocardial segments. Peak global longitudinal strain (GLS) is an average of the longitudinal strain (LS) values obtained from each view and a total of 16 segments (6 basal, 6 midventricular, and 4 apical segments).

**Figure 2:**
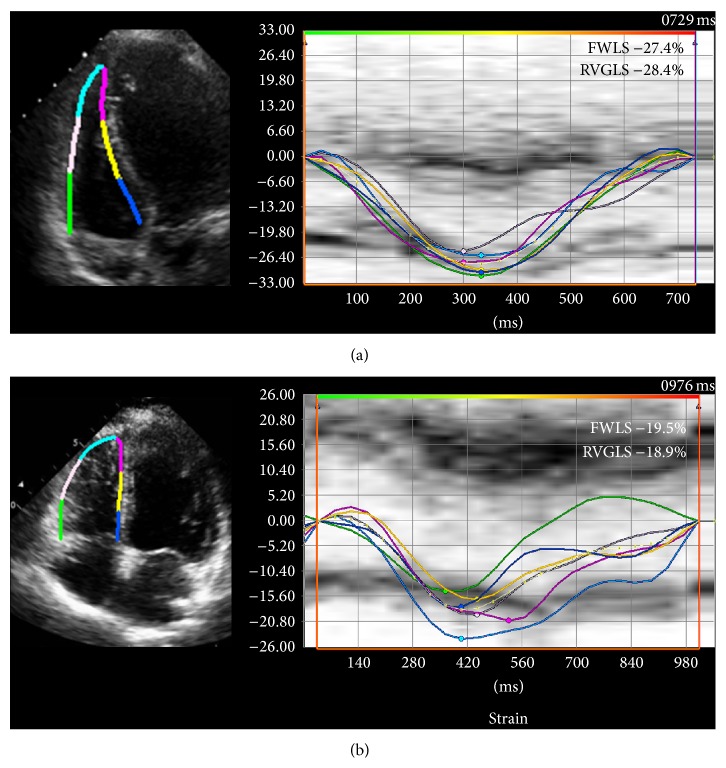
Right ventricular systolic longitudinal strain. Right ventricular peak endocardial strain curves. Left panel shows B-mode images of the RV in 4-chamber view with endocardial tracing and to its right is the corresponding strain curve in (a) control and (b) during cardiotoxicity. Right ventricular free wall longitudinal strain (RVFWLS) is composed of 3 segments while right ventricular peak systolic global longitudinal strain (RVGLS) includes all 6 segments (RV free wall and septum).

Myocardial peak systolic longitudinal strain was measured offline for both the LV and RV based on the ASE recommendations [21] using commercially available software (Vector Velocity Imaging (VVI) 3.0, Siemens Medical Solutions, Mountain View, CA) using the speckle tracking technique. Briefly, apical 4-, 3-, and 2-chamber images of the LV and an apical 4-chamber view of the RV in DICOM format were obtained for each patient and loaded into the VVI software. Endocardial contours were drawn along the LV and RV border separately for measurement of respective longitudinal strain values (Figures 1 and 2). The contours were adjusted as needed to ensure adequate visual tracking of the endocardium. Any segments that were not tracked adequately after 5 attempts at adjustment were excluded from the analysis. LV peak systolic global longitudinal endocardial strain (GLS) was measured by taking the average of the peak endocardial strain curves in the apical 4-, 3-, and 2-chamber views (16-segment model) (Figure 1). As conventionally done, RV peak systolic global longitudinal endocardial strain (RVGLS) was measured from all 6 RV myocardial segments from an apical 4-chamber view (3 segments of the free wall and 3 segments of the interventricular septum) while the RV free wall peak systolic longitudinal strain (RVFWLS) was obtained from the 3 RV free wall segments only (Figure 2). This distinction is made since measurement of RVGLS based on the inclusion of the interventricular septum may partially reflect changes in the left ventricle as the septum is shared by both ventricles. RVFWLS focuses only on the RV free wall and does not include contribution of the septum; however, it does not account for potential changes that may occur in the RV septum.

### 2.4. Follow-Up

As a hypothesis generating objective, to examine the prognostic value of RV dysfunction on subsequent LV function recovery after completion of all cancer therapy, we identified those who had at least one follow-up echocardiogram ≥3 months after completion of their cancer therapy with adequate image quality. In these patients the last available echocardiogram was used to measure LVEF, FAC, and strain values. In addition the following clinical history was obtained through EPR: cardiac symptoms, medication use, and new diagnosis of other cardiac conditions.

### 2.5. Interobserver and Intraobserver Variability

Twenty randomly selected studies were reanalyzed by the same observer (AC) several months after the initial analysis and a second observer (FP) blinded to the original measurements for the assessment of intra- and interobserver variability of RV strain measurements.

### 2.6. Statistical Analysis

Normality for each variable was tested using a combination of quantile-quantile plots and the Kolmogorov-Smirnov test. Depending on the normality, variables are expressed as mean ± SD or median and interquartile range (IQR). Independent sample *t*-test or Wilcoxon rank sum test was used to compare continuous data between groups. A paired *t*-test or a Wilcoxon sign rank test was used to compare patients at the time of cardiotoxicity and at follow-up. Fisher's exact test was used to compare categorical data between the groups. Intraclass correlation coefficient (ICC) was used to assess interobserver and intraobserver variability. A *P* value of <0.05 was considered statistically significant. All statistical analyses were performed using MedCalc software (ver 11.4.2 Belgium).

## 3. Results

### 3.1. Patients and Cancer Treatment

A total of 598 female patients with breast cancer received trastuzumab treatment between 2006 and 2013 at the Princess Margaret Cancer Center. Amongst these patients 30 (5%) were identified as having experienced cardiotoxicity, had a diagnostic quality echocardiogram at the time of cardiotoxicity, and met our inclusion criteria. The remaining 568 patients were either not identified as having cardiotoxicity by the treating oncologist, had an echocardiography study with poor image quality at the time of cardiotoxicity, or were only followed by MUGA studies. Amongst the 30 included patients, 26 (87%) had early stage breast cancer (≤ stage III) and 4 patients had metastatic disease. In patients with early stage disease, 13 received doxorubicin and cyclophosphamide followed by either paclitaxel or docetaxel and TZM (AC-TH) while 9 received 5-fluorouracil, epirubicin, and cyclophosphamide followed by docetaxel and TZM (FEC-DH), and 3 patients received docetaxel, carboplatin or cyclophosphamide, and TZM (TCH). One patient with early stage disease with history of prior early breast cancer treated with FEC twelve years before her new breast cancer diagnosis received only TZM and docetaxel. The 4 patients with metastatic disease received a combination of a taxane (either paclitaxel or docetaxel) and TZM. In patients who received anthracyclines, the mean cumulative dose of epirubicin administered was 302 ± 10 mg/m^2^ while in those who received doxorubicin the mean dose was 231 ± 19 mg/m^2^ (Table 1).

### 3.2. Timing and Management of Cardiotoxicity

All patients with cardiotoxicity had normal pre-chemotherapy LV and RV function by MUGA with a mean LVEF of 62 ± 5% and mean RVEF of 45 ± 5%. In patients who received anthracycline followed by trastuzumab (*n* = 22), cardiotoxicity occurred immediately after anthracycline therapy in one and during trastuzumab treatment in the rest with a median (IQR) time to occurrence of 5.0 (5.0) months. In the 8 patients who received trastuzumab without anthracyclines, the median (IQR) time to cardiotoxicity was 7.5 months (5.7). At the time of cardiotoxicity, 17 (57%) patients reported symptoms consistent with NYHA 2-3, while the rest were NYHA 1. Management of cardiotoxicity included withholding TZM treatment only in 10 patients, adding ACE inhibitors and/or beta-blockers along with withholding TZM in 9 patients, and starting ACE inhibitors and/or beta blockers while continuing TZM in 4. In 7 patients, TZM was continued with close monitoring. The latter reflects the variability in clinical practice.

### 3.3. Ventricular Function at Time of Cardiotoxicity

LV function by MUGA was significantly reduced to 48 ± 4% compared to pretreatment values of 62 ± 5% (*P* < 0.0001). LVEF by MUGA and echo at the time of cardiotoxicity were not significantly different (48 ± 4% versus 46 ± 6%, *P* = 0.13). When compared to controls, the LVEF by echocardiography in patients with cardiotoxicity was significantly lower as expected (59 ± 2% versus 46 ± 6, *P* < 0.0001). Similarly LV peak systolic global longitudinal strain (GLS −21.1 ± 1.2% versus −15.5 ± 2.4%, *P* < 0.0001) was significantly lower in patients with cardiotoxicity compared to controls (Table 2). This value is also significantly lower than the published lower limit of normal for LV GLS of −18.9% (*P* < 0.001) [9, 22].

Precancer treatment RV function by MUGA was 45 ± 4% (normal) [23] while at the time of cardiotoxicity it was 43 ± 6 (*P* = 0.19). None of the patients had abnormal RV function pretherapy. By echocardiography at the time of cardiotoxicity the mean RV function by FAC was significantly lower than the controls although still in the normal range (42 ± 7% versus 47 ± 6% *P* = 0.01) with 3 (10%) patients having an abnormal FAC (<35%). The RV strain was significantly decreased (*i.e., less negative*) in patients with cardiotoxicity compared to controls for both RVGLS (−21.0 ± 3.1% versus −25.7 ± 2.7%, *P* < 0.0001) and RVFWLS (−25.0 ± 4.3% versus −28.8 ± 3.6%, *P* = 0.005) (Table 2). Using a cut-off value of −20.3% for RVGLS and −21.6% for RVFWLS (2SD below mean value for the controls), 12 (40%) and 5 patients (17%), respectively, had reduced RV function by strain analysis. The RVSP (a marker of pulmonary systolic pressures) was also slightly higher in patients with cardiotoxicity compared to controls (Table 2) although still within the normal range. The changes in LV and RV parameters at the time of cardiotoxicity in patients who received anthracycline versus those who did not are summarized in Table 3.

### 3.4. Ventricular Function at Follow-Up

Follow-up echocardiograms at least* 3 months after completion of cancer therapy* were available in 16 of the 30 patients. Mean time from completion of therapy to follow-up echocardiogram was 23 ± 15 months. At follow-up, compared to the time of cardiotoxicity, there was significant improvement in LVEF (from 45 ± 6% to 52 ± 7%, *P* = 0.0001) and LV GLS (−15.2 ± 2.2 to −17.2 ± 2.6%, *P* = 0.004). However, only 5 patients at follow-up had a Biplane LVEF in the normal range (i.e., ≥55%). The mean RV function measured by FAC did not change significantly between the time of cardiotoxicity and follow-up (43 ± 8% to 46 ± 8%, *P* = 0.13). Likewise there was no significant improvement in RVGLS (−20.5 ± 2.6% to −22.9 ± 5% *P* = 0.09) or RVFWLS (−25.0 ± 3.9% to −26.2 ± 5.9% *P* = 0.48) at follow-up. The changes in LV and RV parameters between time of cardiotoxicity and follow-up in patients with LV dysfunction only versus those with coexisting LV and RV dysfunction at the time of cardiotoxicity is summarized in Table 4. Also ventricular function parameters at baseline, at the time of cardiotoxicity, and at follow-up are provided separately for patients with and without LVEF recovery in Table 5.

We also examined the association between RV strain abnormalities at the time of cardiotoxicity and subsequent recovery of LVEF at follow-up. LVEF recovery was seen in only 1 out of 6 patients (17%) with abnormal RVGLS at the time of cardiotoxicity while it was seen in 4 out of 10 patients (40%) with normal RVGLS (*P* = 0.59). Similarly, recovery in LVEF did not occur in any of the 3 patients (0%) with abnormal RVFWLS at the time of cardiotoxicity while it occurred 5 out of 13 patients (38%) with normal RVFWLS (*P* = 0.51). When patients with and without LV function recovery were compared, none of the patients with ventricular function recovery had any cardiac risk factors (diabetes, hypertension, or hypercholesterolemia) while 64% of the patients without recovery had at least 1 cardiac risk factors. There was no difference in mean age (57 ± 24 years versus 56 ± 9 years, *P* = 0.87), mean duration of follow-up (26 ± 14 versus 22 ± 15 *P* = 0.58), the proportion that received anthracyclines (80% versus 73%, *P* = 0.99), and the lowest mean LVEF during cardiotoxicity (49 ± 6% versus 44 ± 6%, *P* = 0.14), between patients with and without recovery, respectively. In the 5 patients with LVEF recovery, 2 were treated with cardiac medications at 1.5 and 4 months from the diagnosis of cardiotoxicity, while in 11 patients without recovery 8 received cardiac medications, with 6 treated at mean of 1.7 ± 1.4 months from diagnosis of cardiotoxicity and 2 patients were treated after 3.5 months due to fluctuating LVEF.

In the remaining 14 out of 30 patients not described above; repeat imaging was done in 11 during the course of cancer treatment but not afterwards, 2 patients had echocardiograms early after treatment completion but were technically inadequate for strain analysis, and one patient was lost to follow up. When these 14 patients were compared to the 16 patients above, there was no statistically significant difference in age (51 ± 14 versus 58 ± 11, *P* = 0.10) or echocardiographic parameters at the time of cardiotoxicity: LVEF (46 ± 6% versus 45 ± 6% *P* = 0.97), GLS (−15.9 ± 2.7% versus −15.2 ± 2.2%, *P* = 0.27), RV FAC (42 ± 6% versus 43 ± 8%, *P* = 0.57), RVFWLS (−25.1 ± 4.8% versus −25.0 ± 3.9%, *P* = 0.68), and RVGLS (−21.6 ± 3.6% versus −20.5 ± 2.6%, *P* = 0.62). In the 13 patients, LVEF at interim follow-up was significantly higher compared to the time of cardiotoxicity (45 ± 6 versus 53 ± 12, *P* = 0.02); however only 7 patients had LVEF >55% at the last available study.

### 3.5. Intraobserver and Interobserver Variability

For the measurement of RVFWLS and RVGLS the intraobserver ICC were 0.97 and 0.97, respectively, while the interobserver ICC were 0.80 and 0.90, respectively.

## 4. Discussion

Our study demonstrates that in women with HER2+ breast cancer that experienced LV cardiotoxicity during treatment with trastuzumab (with or without anthracycline therapy), RV function at the time of cardiotoxicity is lower than controls as measured using FAC (a measure analogous to ejection fraction for the LV) and strain. RV dysfunction was seen in 10% of the patients by FAC and in up to 40% of the patients based on strain analysis by speckle tracking echocardiography. The proportion of patients with abnormal strain was larger than those with abnormal FAC demonstrating the sensitivity of strain measures to identify subtle ventricular dysfunction. In a subgroup of patients with a mean follow-up of 23 months after completion of cancer therapy, LV dysfunction persisted despite appropriate management. Finally, recovery of LV function was lower in patients who had concomitant RV dysfunction at the time of cardiotoxicity compared to those who did not (17% versus 40%); however, this did not reach statistical significance, likely reflecting our small sample size.

### 4.1. Right Ventricular Dysfunction and Cardiotoxicity

In women with HER2+ breast cancer receiving trastuzumab therapy alone or in combination with anthracyclines the incidence of cardiotoxicity (defined by fall in LVEF) in clinical trials has been reported to be as high as 14% [24]. However, in retrospective population based studies the rates are much higher ranging from 15.5 to 41.9% especially in older women and over long term follow-up [25, 26]. The identification of cardiotoxicity has been primarily based on development of LV systolic dysfunction and eventual HF. Currently, RV dysfunction is not considered in the diagnosis of cardiotoxicity and its incidence and prognostic value in patients receiving cancer therapy is unknown. The limited literature on the impact of cancer therapy on the RV may reflect the absence of robust techniques for the assessment of RV function. However, given the thinner structure of the RV with fewer myofibrils, the RV may also be susceptible to damage by cardiotoxic cancer therapy as we have shown in this study. In fact the recent ASE expert consensus statement on the multimodality imaging of adult patients receiving cancer therapy recommends monitoring RV function during cancer therapy [9].

The effect of cancer therapy on the RV was first demonstrated in an older study of 41 doxorubicin treated patients with various cancers where RV wall motion abnormalities were more common than LV abnormalities on radionuclide ventriculography [13]. More recently, a cardiac MRI study of 46 women with breast cancer receiving anthracyclines with or without trastuzumab illustrated RV dysfunction in 34% of the patients by 12 months, while LV dysfunction was seen in 26% [12]. Interestingly RV dysfunction was present as early as 4 months into therapy and was felt to represent an early sign of myocardial injury. Another echocardiography study identified mild reduction in RV FAC and tricuspid annular plane systolic excursion (TAPSE) even as early as the 3rd cycle of doxorubicin therapy in 37 anthracycline treated patients with breast cancer [14]. Two other studies of 19 and 56 survivors of pediatric cancers have shown a reduction in RV free wall strain values (a marker of subclinical ventricular dysfunction) at cumulative anthracycline (various) doses <300 mg/m^2^ [27, 28]. In more recent study of patients with advanced HF receiving LV mechanical circulatory support, patients with chemotherapy induced cardiomyopathy were significantly more likely to also require RV mechanical support [29]. However, these findings have not been consistent amongst studies. Two small studies of patients treated with similar doxorubicin equivalent doses as above studies did not demonstrate RV dysfunction by radionuclide angiography and echocardiography when comparing pre- to posttherapy time points [11, 15]. Also, the incidence of concomitant RV dysfunction* at the time of cardiotoxicity* has not been previously studied.

Our work builds on the existing literature by demonstrating that, in women with HER2+ breast cancer receiving trastuzumab therapy, RV dysfunction is seen* at the time of cardiotoxicity*. When compared to controls, the mean FAC, RVGLS, and RVFWLS were significantly reduced in patients experiencing cardiotoxicity. In addition 10% of the patients had abnormal RV function by FAC. Similarly using a threshold for abnormal strain generated based on the control group; up to 40% of patients had abnormal RVGLS or RVFWS. The higher incidence of RV dysfunction based on strain abnormality reflects the higher sensitivity of this measure for myocardial dysfunction. Ventricular strain is a marker of myocardial deformation and has been used widely for the detection of subclinical cardiotoxicity in many diseases including patients receiving cancer therapy [30]. We demonstrate the use of these measures for the first time for the assessment of RV dysfunction* at the time cardiotoxicity* in patients treated with trastuzumab.

### 4.2. Recovery of Ventricular Function

Generally in trastuzumab treated patients with breast cancer, LV dysfunction that occurs at the time of cardiotoxicity is thought to recover with cessation of trastuzumab [31]. However, this finding has not been universal with some studies demonstrating lack of recovery in LV function in as many as 40% of the patients despite receiving appropriate cardiac therapy [7]. Our study demonstrates that 69% of the patients had persistently abnormal LVEF (<55%) at follow-up, and 3 (19%) remained symptomatic (NYHA ≥ 2). Although the incidence likely exaggerated due to incomplete follow-up, our data suggest that LV dysfunction persists in a significant proportion of patients who experience cardiotoxicity during trastuzumab therapy despite appropriate management. A recent study demonstrated that persistent LV dysfunction in follow-up in patients treated for cancer was associated with higher mortality [32]. Mortality data was not available in our study. Also, interestingly our study demonstrates that a significant proportion of patients with abnormal RV function measured by strain at the time of cardiotoxicity did not have subsequent recovery in LV function at follow-up. This suggests that concomitant RV dysfunction may be a marker of more significant cardiac injury and a potential risk factor for persistent LV dysfunction during follow-up. However, given our small sample size this difference did not reach statistical significance. These findings are hypothesis generating and will need to be confirmed in larger studies.

### 4.3. Limitations

This was a retrospective study from a single center with a relative small sample size. However, the low rates of cardiotoxicity at our center and the fact that cardiac function is generally followed by MUGA as opposed to echocardiography explain our small sample size. Also we did not have baseline echocardiography in our patients with cardiotoxicity to ensure that their LV and RV function were normal pre-cancer therapy and to compare strain and EF values at baseline to the time of cardiotoxicity. However, by MUGA, all of the patients had normal pre-cancer therapy LV and RV function. Furthermore, we used an age and cardiac risk factor balanced control group of HER2+ breast cancer patients who had echocardiography prior to any cancer treatment to account for this limitation. Post-cancer therapy follow-up data was only present in 53% of the patients. This reflects the retrospective nature of the study, and the fact that many patients are not routinely followed with cardiac imaging at our tertiary care center once cancer therapy is completed. Therefore our estimates of ventricular function recovery must be considered in the context of this limitation and is likely higher than expected in clinical practice. We have however shown that the 14 patients without postcancer treatment follow-up were similar to the 16 patients with follow-up with respect to clinical and echocardiographic parameters. We did not include these latter 14 in the follow-up cohort as any conclusion about ventricular function recovery is hampered by ongoing cancer treatment. We also had patients with variability in the cancer treatment. However, all patients received trastuzumab therapy with a majority (73%) also having received anthracyclines. We also did not report measures of TAPSE and systolic annular velocities as additional measures of RV function as this was not consistently available in all the patients. However, we did measure RVGLS in our patients and this has been shown to be a good marker of RV function when compared to the gold-standard of cardiac MRI [33]. Cardiac MRI is considered the reference standard for RV function assessment, but we did not have cardiac MRI data in our patients as this was not standard of care. Finally we did not do a logistic regression analysis of predictors of LV recovery as the number of events was small to meaningfully adjust for confounders.

### 4.4. Clinical Implication

The finding of RV dysfunction during the diagnosis of cardiotoxicity in our study demonstrates the need to assess both ventricles during cancer therapy in patients with breast cancer receiving trastuzumab therapy. This is consistent with recent guidelines from the American Society of Echocardiography, which encourages routine follow-up of both LV and RV functions during cancer treatment [9]. Also, our findings of persistent LV dysfunction during follow-up have implications for cardiac therapy in patients experiencing cardiotoxicity. The appropriate length of treatment with cardiac medications such as beta-blockers and ACE inhibitors in patients experiencing cardiotoxicity during cancer therapy is unknown. Based on our findings of persistently reduced LVEF it may be necessary to continue cardiac treatment for a prolonged period of time. In addition, these patients may need close cardiology follow-up.

## 5. Conclusion

In patients with HER2+ breast cancer treated with trastuzumab with or without anthracyclines who experienced cardiotoxicity (based on reduction in LVEF), concomitant RV dysfunction was seen in up to 40% of the patients based on RV strain measurements. During follow-up after completion of cancer therapy, LV dysfunction (LVEF < 55%) persisted in 69%. Patients with concomitant LV and RV dysfunction at the time of cardiotoxicity had a lower propensity for subsequent ventricular function recovery although this did not reach statistical significance. The prognostic value of RV dysfunction and its persistence during follow-up needs to be assessed in larger studies.

## Figures and Tables

**Table 1:** Patient and control demographics.

	Control *n* = 30	Cohort *n* = 30
Age	51 ± 8	54 ± 12
Stages (I–III)	30 (100%)	26 (87%)
NYHA II-III	—	16 (53%)
Cardiac risk factor		
Coronary artery disease	—	—
Hypertension	5 (17%)	6 (20%)
Diabetes mellitus	1 (3%)	3 (10%)
Dyslipidemia	—	3 (10%)
Smoker	3 (10%)	1 (3%)
Chemotherapeutic regimen		
AC-TH		13 (43%)
Epirubicin mg/m^2^	—	^*^302.4 ± 10
FEC + DH		9 (30%)
Doxorubicin mg/m^2^	—	^*^231.2 ± 18.7
TCH		3 (10%)
TH		5 (17%)
Radiation		23 (77%)
Mastectomy		21 (70%)
Previous cancer^†^		6 (20%)
Ventricular systolic function by MUGA (%)		
LVEF		
Prechemo	—	62 ± 5
At time of cardiotoxicity		48 ± 4
RVEF		
Prechemo		45 ± 4
At time of cardiotoxicity		43 ± 6

^*^Mean cumulative dose, ^†^4 were previously diagnosed with breast tumor and at the time of the study were being treated for recurrence, and 2 had previous history of Ewing's Sarcoma and Hodgkin's lymphoma. AC-TH: doxorubicin and cyclophosphamide followed by either paclitaxel or docetaxel and trastuzumab; FEC-DH: 5-fluorouracil, epirubicin, and cyclophosphamide followed by docetaxel and trastuzumab; TCH: docetaxel, carboplatin or cyclophosphamide, and trastuzumab; TH: trastuzumab and docetaxel or paclitaxel.

**Table 2:** Conventional and strain parameters measured by echocardiography in patients with cardiotoxicity and the control group.

	Control *n* = 30	Cardiotoxicity *n* = 30	*P*
Left ventricle			
EF Biplane (%)	59 ± 2	46 ± 6	<0.0001
GLS	−21.1 ± 1.2	−15.5 ± 2.4	<0.0001
Right ventricle			
RVSP mmHg	24 ± 6.4	29 ± 7.5	0.01
FAC (%)	47 ± 6	42 ± 7	0.01
RVFWLS	−28.8 ± 3.6	−25.0 ± 4.3	0.0005
RVGLS	−25.7 ± 2.7	−21.0 ± 3.1	<0.0001

GLS: global longitudinal strain, FAC: fractional area change, RVFWLS: right ventricular free wall longitudinal strain (3 segments), and RVGLS: right ventricular global longitudinal strain (includes all 6 segments).

**Table 3:** Ventricular function at the time of cardiotoxicity by chemotherapy agent received.

	(+) Anthracycline *n* = 22	(−) Anthracycline *n* = 8
Time to toxicity in months^*^	5 (5)	7.5 (5.7)
Left ventricle		
Baseline EF by MUGA (%)	62 ± 6	60 ± 3
Cardiotoxicity EF by MUGA (%)	48 ± 4	47 ± 4
Cardiotoxicity EF by ECHO (%)	46 ± 6	44 ± 5
Cardiotoxicity GLS	−15.5 ± 2.5	−15.7 ± 2.5
Right ventricle		
Baseline EF by MUGA (%)	46 ± 4	43 ± 3
Cardiotoxicity EF by MUGA (%)	44 ± 6	40 ± 5
Cardiotoxicity FAC by ECHO (%)	42 ± 8	43 ± 5
Cardiotoxicity RVFWLS	−24.1 ± 3.9	−27.4 ± 4.6
Cardiotoxicity RVGLS	−20.4 ± 2.8	−22.8 ± 3.2

^*^Median (IQR) onset of cardiotoxicity which occurred after initiation of TZM therapy in 21 patients; in 1 patient toxicity occurred after 3 months from the start of chemotherapy. Abbreviations as per Table 2.

**Table 4:** Ventricular function at time of cardiotoxicity and at follow-up (*n* = 16) in patients with only left ventricular dysfunction at the time of cardiotoxicity and in those with biventricular dysfunction.

	Cardiotoxicity	Posttreatment
Left ventricular dysfunction *n* = 10		
EF Biplane (%)	44 ± 7	51 ± 9
GLS	−15.7 ± 2.4	−17.6 ± 3.1
FAC (%)	44 ± 5	46 ± 9
RVGLS	−22.2 ± 1.4	−23.2 ± −5.5
RVFWLS	−27.0 ± 3.2	−26.1 ± 6.6
Biventricular dysfunction based on RVGLS *n* = 6		
EF biplane (%)	47 ± 3	52 ± 3
LV GLS	−14.4 ± 1.7	−16.4 ± 1.6
FAC	39 ± 11	48 ± 6
RVGLS	−17.8 ± 1.2	−22.3 ± 2.9
RVFWLS	−21.6 ± 2.8	−26.3 ± 5.0
Biventricular dysfunction based on RVFWLS *n* = 3		
EF biplane (%)	49 ± 5	52 ± 2.5
LV GLS	−15.6 ± 0.8	−16.1 ± 1.1
FAC	33 ± 11	48 ± 3
RVGLS	−14.6 ± 0.7	−21.8 ± 2.1
RVFWLS	−19.5 ± 1.1	−23.5 ± 2.4

Abbreviations as per Table 2.

**Table 5:** Ventricular function parameters at the time of cardiotoxicity and at follow-up (*n* = 16) in patients with and without left ventricular ejection fraction recovery to ≥55%.

	(+) LV recovery n = 5	(−) LV recovery *n* = 11
Left ventricle		
Baseline EF by MUGA (%)	62 ± 8	61 ± 4
Cardiotoxicity EF by MUGA (%)	49 ± 3	45 ± 3
Cardiotoxicity EF by ECHO (%)	49 ± 6	44 ± 6
Post-treatment EF by ECHO (%)	59 ± 2	48 ± 6
Cardiotoxicity GLS	−15.6 ± 2.8	−15.0 ± 1.9
Post-treatment GLS	−19.8 ± 2.3	−16.0 ± 1.8
Right ventricle		
Baseline EF by MUGA (%)	48 ± 3	48 ± 4
Cardiotoxicity EF by MUGA (%)	41 ± 5	42 ± 4
Cardiotoxicity FAC by ECHO (%)	45 ± 3	42 ± 9
Post-treatment FAC by ECHO (%)	54 ± 4	43 ± 8
Cardiotoxicity RVFWLS	−25.3 ± 2.2	−24.8 ± 4.6
Post-treatment RVFWLS	−32.1 ± 5.3	−23.4 ± 3.8
Cardiotoxicity RVGLS	−21.1 ± 1.8	−20.2 ± 2.8
Post-treatment RVGLS	−24.6 ± 3.7	−22.1 ± 4.9

Abbreviations as per Table 2; recovery is defined as an LVEF ≥55% at last follow-up.

## References

[1] American Cancer Society (2014). *Cancer Facts & Figures*.

[2] Canadian Cancer Society's Advisory Committee on Cancer Statistics (2014). *Canadian Cancer Statistics 2014*.

[3] Coleman M. P., Forman D., Bryant H. (2011). Cancer survival in Australia, Canada, Denmark, Norway, Sweden, and the UK, 1995–2007 (the international cancer benchmarking partnership): an analysis of population-based cancer registry data. *The Lancet*.

[4] Su M. Y., Lin L. Y., Tseng Y. H. (2014). CMR-verified diffuse myocardial fibrosis is associated with diastolic dysfunction in HFpEF. *JACC: Cardiovascular Imaging*.

[5] Isner J. M., Ferrans V. J., Cohen S. R. (1983). Clinical and morphologic cardiac findings after anthracycline chemotherapy. Analysis of 64 patients studied at necropsy. *The American Journal of Cardiology*.

[6] Cardinale D., Colombo A., Lamantia G. (2010). Anthracycline-induced cardiomyopathy: clinical relevance and response to pharmacologic therapy. *Journal of the American College of Cardiology*.

[7] Cardinale D., Colombo A., Torrisi R. (2010). Trastuzumab-induced cardiotoxicity: clinical and prognostic implications of troponin I evaluation. *Journal of Clinical Oncology*.

[8] Felker G. M., Thompson R. E., Hare J. M. (2000). Underlying causes and long-term survival in patients with initially unexplained cardiomyopathy. *The New England Journal of Medicine*.

[9] Plana J. C., Galderisi M., Barac A. (2014). Expert consensus for multimodality imaging evaluation of adult patients during and after cancer therapy: a report from the American Society of Echocardiography and the European Association of Cardiovascular Imaging. *Journal of the American Society of Echocardiography*.

[10] Seidman A., Hudis C., Pierri M. K. (2002). Cardiac dysfunction in the trastuzumab clinical trials experience. *Journal of Clinical Oncology*.

[11] Cottin Y., Touzery C., Coudert B. (1996). Diastolic or systolic left and right ventricular impairment at moderate doses of anthracycline? A 1-year follow-up study of women. *European Journal of Nuclear Medicine*.

[12] Grover S., Leong D. P., Chakrabarty A. (2013). Left and right ventricular effects of anthracycline and trastuzumab chemotherapy: a prospective study using novel cardiac imaging and biochemical markers. *International Journal of Cardiology*.

[13] Barendswaard E. C., Prpic H., van der Wall E. E., Camps J. A. J., Keizer H. J., Pauwels E. K. J. (1991). Right ventricle wall motion abnormalities in patients treated with chemotherapy. *Clinical Nuclear Medicine*.

[14] Tanindi A., Demirci U., Tacoy G. (2011). Assessment of right ventricular functions during cancer chemotherapy. *European Journal of Echocardiography*.

[15] Ayhan S. S., Özdemir K., Kayrak M. (2012). The evaluation of doxorubicin-induced cardiotoxicity: comparison of Doppler and tissue Doppler-derived myocardial performance index. *Cardiology Journal*.

[16] Chrysohoou C., Antoniou C.-K., Kotrogiannis I. (2011). Role of right ventricular systolic function on long-term outcome in patients with newly diagnosed systolic heart failure. *Circulation Journal*.

[17] Guendouz S., Rappeneau S., Nahum J. (2012). Prognostic significance and normal values of 2D strain to assess right ventricular systolic function in chronic heart failure. *Circulation Journal*.

[18] Haddad F., Doyle R., Murphy D. J., Hunt S. A. (2008). Right ventricular function in cardiovascular disease, part II: pathophysiology, clinical importance, and management of right ventricular failure. *Circulation*.

[19] Lang R. M., Bierig M., Devereux R. B. (2005). Recommendations for chamber quantification: a report from the American Society of Echocardiography's guidelines and standards committee and the Chamber Quantification Writing Group, developed in conjunction with the European Association of Echocardiography, a branch of the European Society of Cardiology. *Journal of the American Society of Echocardiography*.

[20] Nagueh S. F., Appleton C. P., Gillebert T. C. (2009). Recommendations for the evaluation of left ventricular diastolic function by echocardiography. *Journal of the American Society of Echocardiography*.

[21] Mor-Avi V., Lang R. M., Badano L. P. (2011). Current and evolving echocardiographic techniques for the quantitative evaluation of cardiac mechanics: ASE/EAE consensus statement on methodology and indications endorsed by the Japanese Society of Echocardiography. *Journal of the American Society of Echocardiography*.

[22] Yingchoncharoen T., Agarwal S., Popović Z. B., Marwick T. H. (2013). Normal ranges of left ventricular strain: a meta-analysis. *Journal of the American Society of Echocardiography*.

[23] Pfisterer M. E., Battler A., Zaret B. L. (1985). Range of normal values for left and right ventricular ejection fraction at rest and during exercise assessed by radionuclide angiocardiography. *European Heart Journal*.

[24] Tan-Chiu E., Yothers G., Romond E. (2005). Assessment of cardiac dysfunction in a randomized trial comparing doxorubicin and cyclophosphamide followed by paclitaxel, with or without trastuzumab as adjuvant therapy in node-positive, human epidermal growth factor receptor 2-overexpressing breast cancer: NSABP B-31. *Journal of Clinical Oncology*.

[25] Chen J., Long J. B., Hurria A., Owusu C., Steingart R. M., Gross C. P. (2012). Incidence of heart failure or cardiomyopathy after adjuvant trastuzumab therapy for breast cancer. *Journal of the American College of Cardiology*.

[26] Du X. L., Xia R., Burau K., Liu C.-C. (2011). Cardiac risk associated with the receipt of anthracycline and trastuzumab in a large nationwide cohort of older women with breast cancer, 1998–2005. *Medical Oncology*.

[27] Ganame J., Claus P., Uyttebroeck A. (2007). Myocardial dysfunction late after low-dose anthracycline treatment in asymptomatic pediatric patients. *Journal of the American Society of Echocardiography*.

[28] Yağci-Küpeli B., Varan A., Yorgun H., Kaya B., Büyükpamukçu M. (2012). Tissue Doppler and myocardial deformation imaging to detect myocardial dysfunction in pediatric cancer patients treated with high doses of anthracyclines. *Asia-Pacific Journal of Clinical Oncology*.

[29] Oliveira G. H., Dupont M., Naftel D. (2014). Increased need for right ventricular support in patients with chemotherapy-induced cardiomyopathy undergoing mechanical circulatory support: outcomes from the intermacs registry (interagency registry for mechanically assisted circulatory support). *Journal of the American College of Cardiology*.

[30] Thavendiranathan P., Poulin F., Lim K. D., Plana J. C., Woo A., Marwick T. H. (2014). Use of myocardial strain imaging by echocardiography for the early detection of cardiotoxicity in patients during and after cancer chemotherapy: a systematic review. *Journal of the American College of Cardiology*.

[31] Romond E. H., Jeong J.-H., Rastogi P. (2012). Seven-year follow-up assessment of cardiac function in NSABP B-31, a randomized trial comparing doxorubicin and cyclophosphamide followed by paclitaxel (ACP) with ACP plus trastuzumab as adjuvant therapy for patients with node-positive, human epidermal growth factor receptor 2-positive breast cancer. *Journal of Clinical Oncology*.

[32] Oliveira G. H., Mukerji S., Hernandez A. V. (2014). Incidence, predictors, and impact on survival of left ventricular systolic dysfunction and recovery in advanced cancer patients. *The American Journal of Cardiology*.

[33] Freed B. H., Tsang W., Bhave N. M. (2014). Right ventricular strain in pulmonary arterial hypertension: a 2D echocardiography and cardiac magnetic resonance study. *Echocardiography*.

